# Comparison of Complication Rates Between Transforaminal Interbody Fusion and Anteroposterior Fusion for One- or Two-Level Degenerative Lumbar Spine Surgery: A Propensity Score Matched Analysis

**DOI:** 10.7759/cureus.66455

**Published:** 2024-08-08

**Authors:** Kosei Nagata, Steven D Glassman, Morgan E Brown, Christy Daniels, Patrick Merkel, Mladen Djurasovic, Jeffrey Gum, Leah Carreon

**Affiliations:** 1 Orthopedic Surgery, University of Tokyo Hospital, Tokyo, JPN; 2 Orthopedics, Norton Leatherman Spine Center, Norton Healthcare, Louisville, USA; 3 Research, Norton Leatherman Spine Center, Norton Healthcare, Louisville, USA; 4 Orthopedics, University of Louisville School of Medicine, Louisville, USA

**Keywords:** leg pain, back pain, lumbar fusion, radiculopathy, complications, anterior lumbar fusion, transforaminal interbody fusion

## Abstract

Introduction

Although transforaminal interbody fusion (TLIF) and anterior lumbar interbody fusion (ALIF) combined with posterior fusion (AP) have similar fusion rates, it is unclear if choice of approach has an impact on post-operative complications.

Research question

Is the incidence of residual leg and/or back pain requiring additional treatment after one- or two-level TLIF and AP similar?

Material and methods

Adult patients who underwent one- or two-level TLIF or AP for degenerative pathology were identified and matched using age, sex, body mass index (BMI), American Society of Anesthesiologists (ASA), insurance status, smoking status, revision and number of levels fused. The incidence of radicular leg and back pain requiring emergency department visit/readmission or same level surgical intervention was compared between the two groups.

Results

Of the 319 TLIF and 288 AP cases, 119 cases in each cohort were matched. TLIF patients had shorter operative times (203 min vs 258 min, P<0.001) and hospital stays than the AP patients (3.76 days vs 4.98 days, P<0.001). The incidence of residual leg pain (7 vs 5, P=0.769) and back pain (13 vs 15, P=0.841) was similar between the two groups. Except for constipation, which was more common in the AP group, the incidence of complications was similar between the two groups.

Conclusions

Patients undergoing one- or two-level TLIF showed shorter operative time and hospital stay compared with those undergoing AP. The incidence of leg radiculopathy and back pain was similar between the two groups. Surgeons should consider these findings as part of the decision-making process regarding which approach to use in patients requiring a lumbar interbody fusion.

## Introduction

Interbody fusion has become a standard surgical treatment option for patients with lumbar degenerative disorders undergoing spinal fusion. Two common surgical strategies are transforaminal interbody fusion (TLIF) or anterior lumbar interbody fusion (ALIF) combined with posterior fusion (AP) [1-9]. Both approaches involve removal of the degenerative disc and insertion of an interbody device [6]. Direct foraminal decompression can be achieved with TLIF [2], while ALIF achieves foraminal decompression indirectly through restoration of the disc and foraminal height [10].

Numerous studies have been published showing that both approaches have similar fusion rates [1,8]. Some studies have shown that ALIF is superior in terms of restoration of the disc height and segmental lordosis as it allows for removal of almost all the disc material and the capacity to insert a large interbody device [2,3,7,8,11], while TLIF has lower cost and shorter length of hospitalization [12,13]. However, there is a paucity of literature evaluating the complication profile of these two approaches and it remains unclear if the choice of approach has an impact on post-operative complications. There are anecdotal reports that TLIF leads to a higher incidence of radiculopathy due to the dissection around the exiting nerve root [14,15].

The purpose of this study is to compare the post-operative complications in patients undergoing one- or two-level TLIF versus AP. As our primary outcome, we evaluated the incidence of 30-day, 90-day, and 365-day complications, specifically radicular leg and back pain requiring additional treatment.

## Materials and methods

After receiving Institutional Review Board approval, patients aged 18 years or older who had undergone one- or two-level TLIF or AP followed by posterior fusion for degenerative pathology between January 2016 and December 2020 were identified. Eligible patients were separated into the TLIF group and the AP group. Patients with an underlying diagnosis of tumor, infection, or trauma were excluded. Patients who had ALIF and TLIF performed during the same admission were excluded. Patient demographics, surgical parameters including operative time and blood loss, surgery-associated complications, and post-operative data were collected through a review of electronic medical records.

To minimize bias, propensity matching was performed [16,17]. A propensity score was calculated for each case using binary logistic regression based on age, sex, body mass index (BMI), American Society of Anesthesiologists (ASA) classification, insurance status, smoking, revision, and number of levels. The propensity score is the probability of assignment to one treatment conditional on a subject's measured baseline covariates. Each TLIF case was then matched to an AP case, with the closest propensity score with a caliper of width of 0.2 of the standard deviation of the logit of the propensity score.

The primary outcome was defined as leg or back pain not present prior to surgery requiring additional treatment. Leg pain was defined as radicular pain radiating from the buttock to below knee. Additional treatments included (1) emergency department (ED) visit for pain, (2) epidural injection in the outpatient clinic, or (3) same level surgical intervention. Surgery associated complications within 90 days of the index surgery were also collected as secondary outcomes, including anemia needing transfusion, pulmonary embolism, mortality, surgical site infection, constipation, and urinary retention. All patients were followed up for one year after surgery.

All analyses were performed using SPSS Version 28.0 (IBM Corp., Armonk, NY). Independent t-tests were used to compare continuous variables between the two groups. Fisher exact tests were used to compare the two groups for categorical variables. Threshold p-value was set at <0.05 for statistical significance.

## Results

A total of 607 patients, 319 in the TLIF group and 288 in the AP group, met the inclusion criteria. Propensity matching produced 119 cases in each group. Demographic data are shown in Table 1.

**Table 1:** Comparison of demographic data before and after propensity matching TLIF, transforaminal interbody fusion; AP, anterior and posterior surgery; BMI, body mass index; ASA, American Society of Anesthesiologists classification

	Before matching, total N = 607			After matching, total N = 238		
	TLIF (N = 319)	AP (N = 288)	P-value	TLIF (N = 119)	AP (N = 119)	P-value
Males, N (%)	129 (40%)	130 (45%)	0.251	56 (47%)	57 (48%)	1.000
Age, years, mean (SD)	60.3 (11.0)	55.1 (12.3)	<0.001	57.8 (12.4)	57.1 (12.4)	0.661
BMI, kg/m^2^, mean (SD)	32.5 (7.1)	30.9 (5.5)	0.002	30.9 (6.9)	31.3 (5.2)	0.686
ASA, N (%)						
1	2 (0.6%)	5 (2%)	0.415	2 (2%)	2 (2%)	0.390
2	86 (27%)	83 (29%)		36 (30%)	27 (23%)	
3	225 (71%)	199 (69%)		78 (66%)	89 (75%)	
4	6 (2%)	1 (0.3%)		3 (3%)	1 (1%)	
Public insurance, N (%)	185 (58%)	178 (62%)	0.362	65 (55%)	80 (67%)	0.063
Smoking status, N (%)						
Non-smoker	125 (39%)	101 (35%)	0.006	42 (35%)	40 (34%)	0.514
Former smoker	137 (43%)	104 (36%)		46 (39%)	54 (45%)	
Current smoker	57 (18%)	83 (29%)		31 (18%)	25 (21%)	
Revision, N (%)	140 (44%)	150 (52%)	0.051	47 (39%)	58 (49%)	0.192
Numbers of levels, N (%)						
One	203 (64%)	182 (63%)	0.933	77 (65%)	77 (65%)	1.000
Two	116 (36%)	106 (37%)		42 (35%)	42 (35%)	

In the AP group, 63 patients underwent same day surgeries, and 56 patients underwent staged procedures. Total operative time and post-anesthesia care unit (PACU) time were shorter in the TLIF group compared to the overall AP group (203 ± 61 minutes vs 258 ± 85 minutes, P<0.001, 130 ± 67 minutes vs 159 ± 69 minutes, P=0.001) (Table 2).

**Table 2:** Summary of surgical parameters TLIF, transforaminal interbody fusion; AP, anterior approach combined with posterior instrumentation; PACU, post-anesthesia care unit; EBL, estimated blood loss

	TLIF (N = 119)	AP (N=119)	P-value
Total operation time (minutes), mean (SD)	203.0 (61.1)	258.4 (85.6)	<0.001
Total PACU time (minutes), mean (SD)	130.2 (67.3)	159.3 (69.1)	0.001
Total EBL (mL), mean (SD)	365.0 (229.2)	323.5 (268.0)	0.202
Intraoperative complication, N (%)			
Dural tear	8 (6.7%)	4 (3.4%)	0.375
Iliac vein laceration	0 (0%)	1 (0.8%)	1.000
Hospital stay (days), mean (SD)	3.8 (1.9)	5.0 (2.0)	<0.001

There was no difference in estimated blood loss (365 ± 229 mL vs 323 ± 268 mL, P=0.202). There were no differences in the incidence of dural tears (8 [6.7%] vs 4 [3.4%], p=0.375) or iliac vein laceration between the two groups. Hospital stay was shorter in the TLIF group versus the entire AP group (3.76 days vs 4.98 days, P<0.001) even after excluding 56 patients who underwent staged surgeries (3.76 days in TLIF, 4.52 days in AP same day, and 5.50 days in AP staged surgery, P<0.001). In 56 patients undergoing staged surgery, the average interval between AP surgery and posterior fusion was 1.5 days (one day in 36 patients, two days in 15 patients, three days in two patients, and four days in three patients).

The incidence of post-operative radicular leg (7 vs 5, P=0.769) and back pain (13 vs 15, P=0.841) requiring an intervention was similar between the two groups at 30, 90, and 365 days after surgery (Table 3).

**Table 3:** Comparison of pain-related post-operative complications between the two groups. TLIF, transforaminal interbody fusion; AP, anterior approach combined with posterior instrumentation; ED, emergency department; CT, computed tomography

	TLIF (N = 119)	AP (N=119)	P-value
Leg pain	7 (5.9%)	5 (4.2%)	0.769
1-30 days post-operative	4 (3.4%)	4 (3.4%)	1.000
31-90 days post-operative	1 (0.8%)	0 (0%)	1.000
91-365 days post-operative	2 (1.6%)	1 (0.8%)	1.000
Back pain	13 (10.9%)	15 (12.6%)	0.841
1-30 days post-operative	8 (6.7%)	5 (4.2%)	0.570
31-90 days post-operative	1 (1.6%)	1 (0.8%)	1.000
91-365 days post-operative	4 (3.4%)	9 (7.6%)	0.253
ED visit within 365 days post-operative (any reason)	17 (14.2%)	21 (17.6%)	0.569
Any type of injection	7 (5.9%)	12 (10.1%)	0.339
Epidural injection	4 (3.4%)	5 (4.2%)	1.000
CT myelogram	7 (5.9%)	3 (2.5%)	0.333
Dorsal column spinal stimulator	2 (1.7%)	1 (0.8%)	1.000

There were seven patients in the TLIF group who complained of leg pain (four had pain on the same side as the TLIF approach side within 30 days after surgery and three had pain on the contralateral TLIF side more than 30 days after surgery). There were five patients with leg pain in the AP group (leg pain was on the right side in two cases, on the left side in one case, and bilaterally in two cases). In both groups, radicular leg pain resolved within 90 days of surgery, with only two patients in the TLIF group and one patient in the AP group reporting leg pain past 90 days post-operative. In 12 of the patients with leg pain, 10 patients also had back pain. In total 30 patients with leg and/or back pain, 19 patients needed ED visit for pain and nine patients needed epidural injection (one needed both ED visit and epidural injection in clinic). The other three patients underwent foraminotomy or seroma removal at the index level.

There were no significant differences between the TLIF group and the AP group in the incidence of pulmonary embolism (2 vs 1), mortality (1 vs 1), surgical site infection (8 vs 6), reoperation (8 vs 6), or urinary retention (8 vs 4) (Table 4). Although the difference in severe constipation between the two groups was not significant (1 vs 7, P=0.066), there were less patients in the TLIF group who had constipation requiring intervention by hospitalists or gastroenterologists than the AP group (14 vs 34, P=0.002).

**Table 4:** Comparison of post-operative complications between the two groups. TLIF, transforaminal interbody fusion; AP, anterior approach combined with posterior instrumentation; SSI, surgical site infection; I&D, irrigation and debridement Severe constipation is defined as requiring an enema.

	TLIF (N = 119)	AP (N=119)	P-value
Anemia needing transfusion	4 (3.4%)	5 (4.2%)	1.000
Pulmonary embolism	2 (1.7%)	1 (0.8%)	1.000
Mortality	1 (0.8%)	1 (0.8%)	1.000
Cumulative SSI	8 (6.7%)	6 (5.0%)	0.784
Minor SSI (without I&D)	4 (3.4%)	4 (3.4%)	1.000
Cumulative reoperation	12 (10.1%)	6 (5.0%)	0.219
SSI requiring I&D	4 (3.4%)	2 (1.7%)	0.683
30-day reoperation excluding SSI	2 (1.7%)	1 (0.8%)	1.000
365-day reoperation excluding SSI	6 (5.0%)	3 (2.5%)	0.499
Cumulative constipation	14 (11.8%)	34 (28.6%)	0.002
Severe constipation	1 (0.8%)	7 (5.9%)	0.066
Mild constipation	13 (10.9%)	27 (22.7%)	0.023
Urinary retention	8 (6.7%)	4 (3.4%)	0.375

## Discussion

Interbody fusion for lumbar degenerative surgeries can be performed through an anterior or posterior approach, each with advantages and disadvantages. Anecdotally, surgeons have concerns for radiculopathy, especially on the ipsilateral side of the TLIF approach [6,14,15]. However, in this propensity-matched study, both approaches had a similar rate of radicular leg pain that required additional treatment. In addition, radicular leg pain was observed on the contralateral side of the TLIF almost as often as on the ipsilateral side. Ipsilateral leg pain may be explained by intraoperative nerve root traction or during dissection. Contralateral leg pain similarly be explained as laminotomies and foraminotomies are often performed on the side opposite the TLIF approach. If a laminectomy is not performed on the contralateral side, leg pain may result from asymmetric entry of TLIF cage and narrowing of the contralateral foramen. Newer surgical techniques such as expandable cages may have led to the lower incidence of radicular leg pain with either approach.

Studies of lower extremity pain in ALIF and TLIF are still insufficient. Although Mobbs et al. [6] argued that ALIF and TLIF had similar visual analog scale for back/leg pain at two years follow-up, there was no analysis from the viewpoint of pain requiring additional intervention. Divi et al. [3] conducted a retrospective cohort study and showed that patients undergoing ALIF had significantly lower preoperative visual analog scale (VAS) leg scores compared to patients undergoing TLIF or posterior lumbar interbody fusion; however, these differences resolved one year after surgery. Their study did not investigate laterality of leg pain.

Although some studies have reported that surgical complications were higher in patients undergoing in TLIF compared to patients undergoing AP [2,14,15] this propensity-matched study revealed that post-operative complications were similar between the two groups except for constipation. Perhaps, this higher incidence of constipation in AP cases can be expected as the surgical approach involves direct manipulation of the bowels. A constipation rate of more than twofold shown in this study may narrow the indications of AP for certain patients, such as the elderly as well as those with a history of abdominal surgery.

Although several studies have shown that TLIF and AP result in similar clinical outcomes [1,3,4,7], AP has been shown to provide better restoration of lumbar lordosis than TLIF [9,11]. However, this advantage of TLIF should be considered by spine surgeons in light of the findings of this study. The longer operative times and length of hospitalization in AP surgery compared to TLIF may be worthwhile for patients who require restoration of alignment. This difference is minimized if the anterior and posterior approaches are performed on the same day. This finding is similar to multicenter studies comparing TLIF and AP surgeries, supporting our results indicating that TLIF had shorter operation time and hospitalization length [2,7].

There are limitations to this study. First, this study set the primary outcome requiring additional medical cost and did not include a measure of the severity of the leg pain, such as a numeric rating scale or VAS for pain. We defined leg or back pain in this study as radicular pain requiring additional treatment. Pain complaints that did not require any intervention were excluded. Second, although several surgeons performed the surgeries, this study constitutes a single center’s experience. Third, the study is retrospective and complications may have been unreported and the potential risk of selection bias still cannot be totally excluded.

## Conclusions

In the current study, patients undergoing TLIF for one- or two-level lumbar fusion surgery for degenerative disorders had shorter operative times and hospital length of stay compared to those undergoing AP following posterior fusion. The incidence of complications including radiculopathy and back pain was similar between the two groups. Surgeons should consider these findings as part of the decision-making process regarding which approach to use in patients requiring a lumbar interbody fusion.
